# Poor neutralization and rapid decay of antibodies to SARS-CoV-2 variants in vaccinated dialysis patients

**DOI:** 10.1371/journal.pone.0263328

**Published:** 2022-02-10

**Authors:** Jessica Bassi, Olivier Giannini, Chiara Silacci-Fregni, Laura Pertusini, Paolo Hitz, Tatiana Terrot, Yves Franzosi, Francesco Muoio, Christian Saliba, Marcel Meury, Exequiel A. Dellota, Josh R. Dillen, Patrick Hernandez, Nadine Czudnochowski, Elisabetta Cameroni, Nicola Beria, Mariangela Ventresca, Alberto Badellino, Soraya Lavorato-Hadjeres, Elisabetta Lecchi, Tecla Bonora, Matteo Mattiolo, Guido Trinci, Daniela Garzoni, Giuseppe Bonforte, Valentina Forni-Ogna, Davide Giunzioni, Lorenzo Berwert, Ravindra K. Gupta, Paolo Ferrari, Alessandro Ceschi, Pietro Cippà, Davide Corti, Antonio Lanzavecchia, Luca Piccoli

**Affiliations:** 1 Humabs BioMed SA, A Subsidiary of Vir Biotechnology, Bellinzona, Switzerland; 2 Faculty of Biomedical Sciences, Università della Svizzera italiana, Lugano, Switzerland; 3 Department of Medicine, Ente Ospedaliero Cantonale, Bellinzona, Switzerland; 4 Division of Nephrology, Ente Ospedaliero Cantonale, Lugano, Switzerland; 5 Clinical Trial Unit, Ente Ospedaliero Cantonale, Lugano, Switzerland; 6 Vir Biotechnology, San Francisco, California, United States of America; 7 Cambridge Institute of Therapeutic Immunology & Infectious Disease (CITIID), Cambridge, United Kingdom; 8 Department of Medicine, University of Cambridge, Cambridge, United Kingdom; 9 Clinical School, University of New South Wales, Sydney, Australia; 10 Division of Clinical Pharmacology and Toxicology, Institute of Pharmacological Science of Southern Switzerland, Ente Ospedaliero Cantonale, Lugano, Switzerland; 11 Department of Clinical Pharmacology and Toxicology, University Hospital Zurich, Zurich, Switzerland; 12 Faculty of Medicine, University of Zurich, Zurich, Switzerland; Waseda University: Waseda Daigaku, JAPAN

## Abstract

Patients on dialysis are at risk of severe course of SARS-CoV-2 infection. Understanding the neutralizing activity and coverage of SARS-CoV-2 variants of vaccine-elicited antibodies is required to guide prophylactic and therapeutic COVID-19 interventions in this frail population. By analyzing plasma samples from 130 hemodialysis and 13 peritoneal dialysis patients after two doses of BNT162b2 or mRNA-1273 vaccines, we found that 35% of the patients had low-level or undetectable IgG antibodies to SARS-CoV-2 Spike (S). Neutralizing antibodies against the vaccine-matched SARS-CoV-2 and Delta variant were low or undetectable in 49% and 77% of patients, respectively, and were further reduced against other emerging variants. The fraction of non-responding patients was higher in SARS-CoV-2-naïve hemodialysis patients immunized with BNT162b2 (66%) than those immunized with mRNA-1273 (23%). The reduced neutralizing activity correlated with low antibody avidity. Patients followed up to 7 months after vaccination showed a rapid decay of the antibody response with an average 21- and 10-fold reduction of neutralizing antibodies to vaccine-matched SARS-CoV-2 and Delta variant, which increased the fraction of non-responders to 84% and 90%, respectively. These data indicate that dialysis patients should be prioritized for additional vaccination boosts. Nevertheless, their antibody response to SARS-CoV-2 must be continuously monitored to adopt the best prophylactic and therapeutic strategy.

## Introduction

Patients with chronic kidney disease (CKD), in particular those with end-stage kidney disease (ESKD) on dialysis, are highly predisposed to infections, which are the major cause of morbidity and the second cause of mortality in this vulnerable population [1, 2]. Infection by SARS-CoV-2, the causative agent of the Coronavirus disease-2019 (COVID-19), has posed a new threat for CKD patients, who were found to have a greater risk of severe COVID-19 course [3, 4]. Early reports indicated a case fatality ranging from 10 to 30% in patients on hemodialysis (HD) [5–10].

The rapid development of COVID-19 vaccines has provided an important strategy to prevent SARS-CoV-2 infection and, especially, severe COVID-19 course. In particular, mRNA-based vaccines developed by Pfizer/BioNTech (BNT-162b2) and Moderna (mRNA-1273) have demonstrated high safety and efficacy in healthy and at-risk individuals, including patients with chronic diseases, cancer and solid organ transplantation [11–14]. However, immunosuppressed patients, in particular those with hematological malignancies, autoimmune diseases and solid organ transplantations, were shown to mount a low antibody response to these vaccines [15–20]. Because of their immunological frailty, patients on dialysis were prioritized in international COVID-19 vaccination programs [21]. Recent studies in HD patients showed a delayed and lower serological response to vaccines and a rapid decline of anti-SARS-CoV-2 antibodies [19, 22–24]. These findings suggest an overall diminished vaccine response to SARS-CoV-2 in ESKD patients that is reminiscent of the low response observed after vaccination against Hepatitis B virus (HBV) and seasonal Influenza virus [25, 26]. In addition, the rapid increase in cases of infection by SARS-CoV-2 Delta variant (B.1.617.2 lineage) since April 2021 provided a new potential challenge for dialysis patients, especially those with a suboptimal response to the vaccine [27].

At this stage of the pandemic, it is urgent to identify populations not developing sufficient levels of neutralizing antibodies against circulating SARS-CoV-2 variants, which therefore may be at-risk of developing severe COVID-19. In this study, we provide evidence of a poor neutralizing and rapidly decaying antibody response to mRNA-vaccines in the dialysis population, which supports the strategy of prioritizing these patients for an additional boost and other therapeutic strategies.

## Material and methods

### Study participants and ethics statement

Blood samples were obtained from 143 dialysis patients and 48 healthcare workers under study protocols approved by the local Institutional Review Board (Canton Ticino Ethics Committee, Switzerland). All the participants were recruited from the four public hospitals of the Ente Ospedaliero Cantonale (EOC) in Ticino (Southern Switzerland). All subjects provided written informed consent for the use of blood and blood components (such as PBMCs, sera or plasma).

### Isolation of plasma

Blood samples were collected from all the participants before, 2–3 weeks and up to 7 months after the second dose. An additional sample was collected 2–3 weeks after the first COVID-19 vaccine dose from 26 patients and all the 48 healthcare workers. Plasma was isolated from blood draw performed using BD tubes containing Ficoll (BD, CPT Ficoll, Cat. No. 362780) and stored at +4°C until use.

### Cell lines

Cell lines used in this study were obtained from ATCC (Vero E6 TMPRSS2) or ThermoFisher Scientific (Expi CHO cells, Expi293F™ and HEK293F cells). Vero E6 TMPRSS2 cells were grown in DMEM supplemented with 10% HyClone (FBS). Expi293F cells were grown in Expi293 Expression Medium.

### Production of recombinant glycoproteins

The SARS-CoV-2 RBD WT construct was synthesized by GenScript into phCMV1, with a sequence encoding an N-terminal mu-phosphatase signal peptide, an ‘ETGT’ linker, SARS-CoV-2 S residues 328–531, a linker sequence, an Avi tag, a twin Strep tag and a 8xHis-tag. Recombinant ACE2 (UniProt Q9BYF1, residues 19–615 with a C-terminal thrombin cleavage site-TwinStrep-10xHis-GGG-tag, and N-terminal signal peptide) and RBD WT constructs were transiently transfected into Expi293F cells following manufacturer’s instructions. Supernatants were clarified by centrifugation and affinity purified using StrepTrap HP columns (Cytiva). Monomeric ACE2 was isolated by size exclusion chromatography using a Superdex 200 Increase 10/300 GL column (Cytiva). The SARS-CoV-2 stabilized Spike WT (D614G) construct was synthesized by GenScript into pCDNA3.1 with an N-terminal mu-phosphatase signal peptide, 2P stabilizing mutation [28, 29], a TEV cleavage site and a C-terminal foldon, 8x His-tag, Avi tag and C-tag [30] and expressed in HEK293F 293 cells following manufacturer’s instructions. Supernatants were clarified by centrifugation and affinity purified using a 5 mL C-tag affinity matrix column.

### VSV spike mutants and pseudovirus generation

Amino acid substitutions were introduced into the D614G pCDNA_SARS-CoV-2_S plasmid as previously described [31]. SARS-CoV-2 S glycoprotein-encoding-plasmids used to produce SARS-CoV-2 VSV, namely the Wuhan prototype (D614, referred as WT) and variants Alpha (B.1.1.7), Beta (B.1.351), Gamma (P.1), Delta (B.1.617.2), Epsilon (B.1.429), Kappa (B.1.617.1) and Lambda (C.37), were obtained using a multistep based on overlap extension PCR (oePCR) protocol [32].

Replication defective VSV pseudovirus [33] expressing SARS-CoV-2 Spike proteins corresponding to the different VOC were generated as previously described [34].

### Plasma pseudovirus neutralization assay

Vero E6-TMPRSS2 were grown in DMEM supplemented with 10% FBS and seeded into white bottom 96 well plates (PerkinElmer, 6005688), as previously described [35]. Conditions were tested in duplicate wells in each plate and at least six wells per plate contained untreated infected cells (defining the 0% of neutralization, “MAX RLU” value) and infected cells in the presence of S2E12 and S2X259 mAbs at 50 μg/mL each (defining the 100% of neutralization, “MIN RLU” value). Average of Relative light units (RLUs) of untreated infected wells (MAX RLU_ave_) was subtracted by the average of MIN RLU (MIN RLU_ave_) and used to normalize percentage of neutralization of individual RLU values of experimental data according to the following formula: (1-(RLU_x_—MIN RLU_ave_) / (MAX RLUave−MIN RLU_ave_)) x 100. Data were analyzed and visualized with Prism (Version 9.1.0). Each neutralization experiment included a technical duplicate. The loss or gain of neutralization potency across Spike variants was calculated by dividing the variant ID_50_ by the parental ID_50_.

### Enzyme-linked immunosorbent assay (ELISA)

Spectraplate-384 with high protein binding treatment (custom made from Perkin Elmer) were coated overnight at 4°C with 5 μg/mL RBD or 1 μg/mL SARS-CoV-2 S protein in PBS. The day after plates were washed and blocked with Blocker Casein in PBS (Thermo Fisher Scientific, 37528) supplemented with 0.05% Tween 20 (Sigma Aldrich), 1h RT. Serial dilutions of plasma samples were then added to plates for 1h RT. Alkaline Phosphatase-conjugated goat anti-human IgG, IgM or IgA (Southern Biotech) were added to plates and incubated for 1h RT. 4-NitroPhenyl Phosphate (pNPP, Sigma-Aldrich, N2765-100TAB) substrate was then added and plates were read after 1 h (IgG) or 2 h (IgA and IgM) at 405 nm with a BioTek plate reader. Data were plotted and analyzed with GraphPad Prism software (version 9.1.0). For chaotropic ELISA, after incubation with plasma, plates were washed and incubated with a 1 M solution of sodium thiocyanate (NaSCN, Sigma 251410) for 1h RT. Avidity Index was calculated as the ratio (%) of the ED_50_ in presence and the ED_50_ in absence of NaSCN.

### Blockade of RBD binding to human ACE2

Plasma were diluted in PBS and mixed with SARS-CoV-2 RBD mouse Fc-tagged antigen (Sino Biological, 40592-V05H, final concentration 20 ng/mL) and incubated for 30 min at 37°C. The percentage of inhibition was calculated as follows: (1−(OD sample−OD neg ctr)/(OD pos ctr−OD neg ctr)]) × 100.

### Statistical analysis

The study was designed to have 80% power to detect a minimum 25% difference in total incidence of cases with poor neutralizing antibody response (i.e., low or undetectable plasma antibody titers) or in average neutralizing titers between dialysis patients and healthy controls as well as within the HD subgroups. Comparisons of means between two groups of unpaired data were made with Mann-Whitney rank test. Comparisons of means between multiple groups of unpaired data were made with Kruskal-Wallis rank test and corrected with Dunn’s test. Comparisons of means between multiple groups of matched data were made with Friedman rank test and corrected with Dunn’s test. Relative risks of poor neutralizing response in selected groups of patients were calculated from 2x2 contingency tables using two-sided Fisher’s exact test. Statistical significance is set as P<0.05 and P-values are indicated with: ns = non-significant; * = 0.033; ** = 0.002; ***<0.001. ED50 and ID50 titers were calculated from the interpolated value from the log(agonist) and the log(inhibitor), respectively, versus response, using variable slope (four parameters) non-linear regression. Data were plotted and analyzed with GraphPad Prism software (version 9.1.0).

## Results

### Naïve dialysis patients produce low or undetectable levels of antibodies after two vaccine doses

At the beginning of the COVID-19 vaccination campaign in January 2021, 143 dialysis patients (130 HD, of whom 68% on hemodiafiltration, and 13 on peritoneal dialysis, PD) were enrolled in this study from four dialysis units of the Ente Ospedaliero Cantonale in Ticino, Switzerland, and received two doses of SARS-CoV-2 mRNA vaccines (83% with BNT162b2 from Pfizer-BioNTech and 17% with mRNA-1273 from Moderna) between January and May 2021. Socio-demographic data, dialysis features, comorbidities, therapies and information about previous vaccinations and infections are summarized in **Table 1**. Of note, twenty-four (17%) dialysis patients were previously infected with SARS-CoV-2, of whom 18 (75%) had severe COVID-19 requiring hospitalization (13) or admission to an intensive care unit (5). A group of 48 healthcare workers, who received two doses of BNT162b2 vaccine between April and June 2021, were included as healthy controls (HC). Twenty-four HC were previously infected with SARS-CoV-2 reporting mild symptoms that did not require hospitalization.

**Table 1:** Socio-demographic and clinical data of enrolled dialysis patients and healthy controls.

	Dialysis patients	Hemodialysis (HD)	Peritoneal dialysis (PD)	Healthy controls (HC)
Enrolled participants	143	130	13	48
Age, median years (min-max, IQR)	76 (29–97, 67–83)	76 (29–97, 68–83)	71 (32–82, 55–78)	42 (24–67, 32–49)
Gender, female, n (%)	48 (34%)	43 (33%)	5 (38%)	35 (73%)
BMI, median Kg/m^2^ (min-max, IQR)	25.9 (17.6–58.8, 22.9–29.2)	26.0 (17.6–58.8, 22.8–29.4)	25.7 (18–36.8, 23.3–29.0)	25.0 (17–3, 21–28)
Smoker, n (%)	21 (15%)	18 (14%)	3 (23%)	13 (27%)
Dialysis treatment, n (%)				
HD, n (%)	130 (91%)	130 (100%)		
HDF, n (%)		89 (68%)		
PD, n (%)	13 (9%)		13 (100%)	
Dialysis vintage, median months (min-max, IQR)	42 (11–268, 25–80)	46 (11–268, 26–87)	28 (11–70, 16–44)	
Dialysis access, native AVF, n (%)	96 (67%)	96 (74%)		
Comorbidities, n (%)				
Diabetes	58 (41%)	56 (43%)	2 (15%)	2 (4%)
Hypertension	121 (85%)	109 (84%)	12 (92%)	5 (10%)
Heart failure	19 (13%)	18 (14%)	1 (8%)	-
Coronary heart disease	46 (32%)	41 (32%)	5 (38%)	-
Peripheral arterial disease	29 (20%)	28 (22%)	1 (8%)	-
Pulmonary disease	42 (29%)	38 (29%)	4 (31%)	-
Chronic liver disease	12 (8%)	12 (9%)	-	-
Gastrointestinal disease	27 (19%)	24 (18%)	3 (23%)	-
Hemato-oncological disease	12 (8%)	12 (9%)	1 (8%)	-
Autoimmune disease	11 (8%)	9 (7%)	2 (15%)	1 (3%)
Kidney transplant	10 (7%)	10 (8%)	-	-
Neurodegenerative disease	4 (3%)	4(3%)	-	-
Psychiatric disease	13 (9%)	13 (10%)	-	-
Charlson comorbidity index, median (min-max, IQR)	8 (3–21, 7–11)	9 (3–21, 7–11)	6 (5–11, 6–8)	-
Previous SARS-CoV-2 infection^1^, n (%)	24 (17%)	23 (18%)	1 (8%)	24 (50%)
mild or no symptoms	6 (25%)	5 (22%)	1 (100%)	24 (50%)
severe course with hospitalization	18 (75%)	18 (78%)	-	-
ICU admission	5 (28%)	5 (28%)	-	-
Diagnosis-vaccine interval, median months (min-max, IQR)	3.5 (1.1–9.7, 2.9–7.7)	3.3 (1.1–9.7, 2.9–7.7)	9.3	4.8 (3.5–12.8, 4.7–5.5)
COVID-19 vaccine received				
Pfizer-BioNTech, n (%)	118 (83%)	107 (82%)	11 (85%)	48 (100%)
Moderna, n (%)	25 (17%)	23 (18%)	2 (15%)	-
Dose 1—dose 2 interval, median days (min-max, IQR)	28 (20–38, 26–28)	28 (20–28, 26–28)	24 (21–28, 22–28)	38 (23–31, 28–28)
Sampling after dose 1, median days (min-max, IQR)	15 (9–33, 13–26)	15 (9–33, 13–26)	-	13 (12–16, 12–14)
Sampling after dose 2, median days (min-max, IQR)	18 (13–27, 17–19)	18 (13–27, 17–19)	18 (16–25, 17–21)	13 (10–21, 12–14)
2020 seasonal flu vaccination, n (%)	101 (70%)	94 (72%)	7 (54%)	29 (60%)
Pharmacological treatment				
Oral anticoagulation	27 (19%)	25 (19%)	2 (15%)	-
Antiaggregant drugs	124 (43%)	124 (95%)	-	-
ACE inhibitors/ARBs	70 (49%)	58 (45%)	12 (92%)	3 (6%)
Calcium antagonists	61 (43%)	51 (39%)	10 (77%)	-
Beta blockers	88 (62%)	79 (61%)	9 (69%)	2 (4%)
Statins	80 (56%)	71 (55%)	9 (69%)	2 (4%)
Oral antidiabetic drugs	22 (15%)	22 (17%)	-	2 (4%)
Insulin	37 (26%)	35 (27%)	2 (15%)	-
Immunosuppressive drugs^2^	24 (17%)	22 (17%)	2 (15%)	-
Oral/intravenous steroids	17 (12%)	16 (12%)	1 (8%)	-
Calcineurin inhibitors^3^	8 (6%)	8 (6%)	-	-
Vitamin D derivatives	112 (78%)	102 (78%)	10 (77%)	-

IQR, interquartile range. BMI, body mass index. HDF, hemodiafiltration. AVF, arteriovenous fistula. PCR, polymerase chain rection. ICU, intensive care unit. ACE, angiotensin-converting enzyme. ARB, angiotensin receptor blockers.

^1^with positive PCR and/or serology.

^2^include any of steroids, cyclosporine, tacrolimus, rituximab, mycophenolate mofetil and azathioprine.

^3^include cyclosporine and tacrolimus.

**Fig 1:**
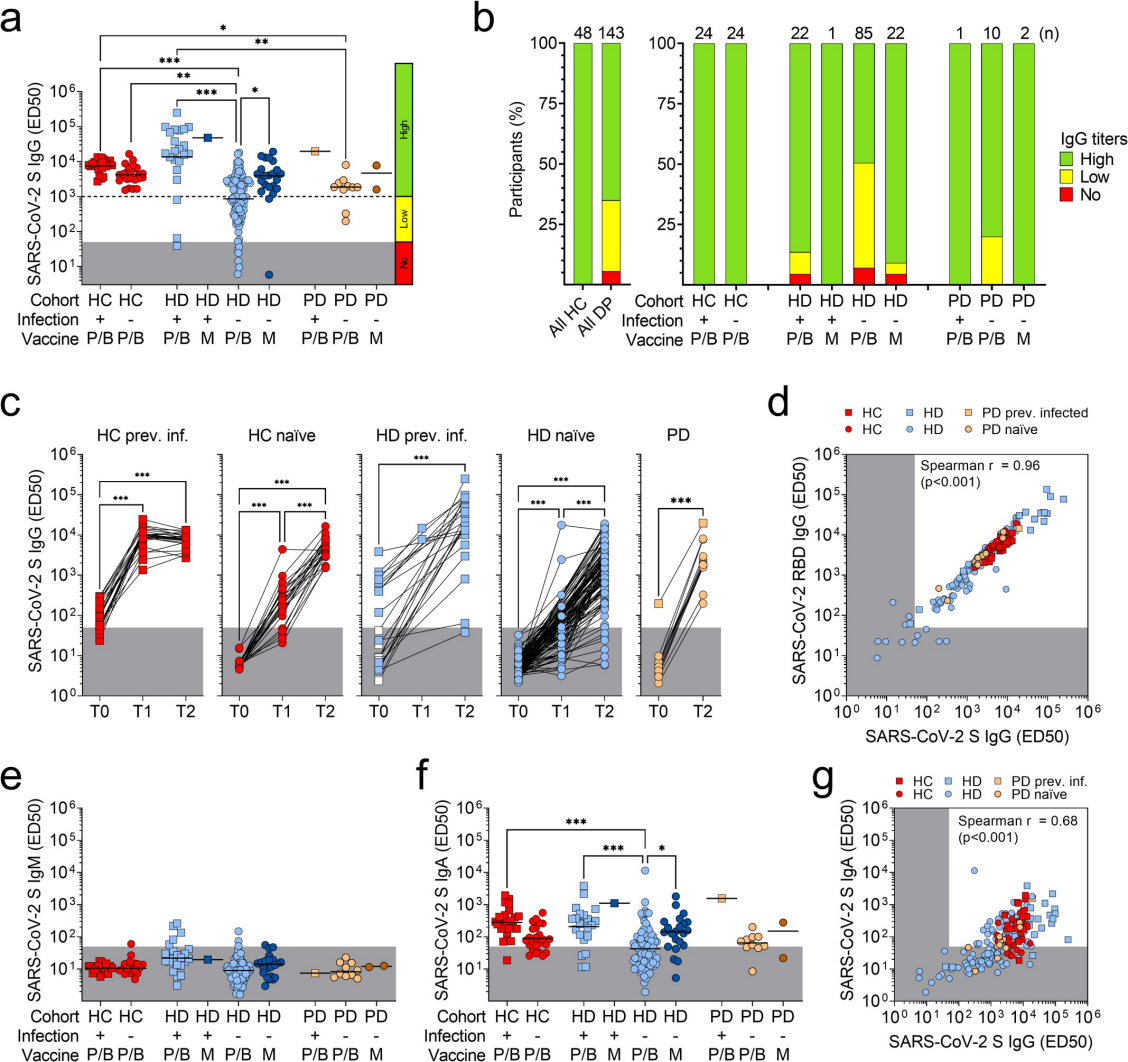
Comparison of mRNA vaccine-induced plasma antibody titers against SARS-CoV-2 between healthy controls and dialysis patients. a) Plasma IgG titers (ED50) to SARS-CoV-2 S after two doses of Pfizer/BioNTech (P/B) or Moderna (M) vaccines in previously infected (square) and naïve (circle) healthy controls (HC, red), hemodialysis (HD, blue) and peritoneal dialysis (PD, orange) patients. Grey areas indicate non-specific IgG titers <50, a cut-off that was determined on non-specific binding to uncoated ELISA plates. An additional cut-off of 1’000, determined from the lowest titers in HC after two doses, was used to distinguish low (50–1’000) from high (>1’000) IgG titers. Statistical significance is set as P<0.05 and P-values are indicated with asterisks (* = 0.033; ** = 0.002; ***<0.001). b) Percentages of participants with high, low or no plasma SARS-CoV-2 S-specific IgG after two doses of mRNA-vaccine. Total number of participants within each cohort of HC and dialysis patients (DP) and within each subgroup is shown at the top of each bar. c) Kinetics of plasma IgG titers to SARS-CoV-2 S measured before vaccination (T0), after one (T1) or two (T2) vaccine doses. Each line connects samples from the same individual. White squares indicate HD patients who were not yet infected at T0 sampling. d) Correlation analysis between plasma IgG titers to SARS-CoV-2 S and RBD in all the plasma samples collected after the second vaccine dose. e) Plasma IgM titers to SARS-CoV-2 S after two vaccine doses in HC, HD and PD patients. Grey areas indicate non-specific IgM titers <50. f) Plasma IgA titers to SARS-CoV-2 S after two vaccine doses in HC, HD and PD patients. Grey areas indicate non-specific IgA titers <50. g) Correlation analysis between plasma IgG and IgA titers to SARS-CoV-2 S in all the plasma samples collected after the second vaccine dose.

Plasma samples were collected 2 to 3 weeks after the first and the second dose and the levels of plasma IgG, IgA and IgM specific for SARS-CoV-2 Spike (S) were measured by ELISA. A single vaccine dose induced detectable IgG in 34.6% of dialysis patients compared to 89.6% of HC, with high antibody levels in all HD and HC participants, who had been previously infected with SARS-CoV-2, and in 29.2% of HD and 79.2% of HC, who had not been exposed to the virus (**S1 Fig**). Importantly, although seroconversion was observed in 94.4% of dialysis patients after the second vaccine dose, 35% of them had still low or undetectable levels of anti-SARS-CoV-2 S antibodies, compared to 100% of HC showing high levels of S-specific IgG (**Fig 1**A and **1**B).

The larger fraction of participants with low or no antibodies (36.9%) was represented by the HD group, with naïve patients immunized with BNT162b2 showing the lowest levels of plasma IgG (50.6%) compared to those who received mRNA-1273 (9.1%) (**Fig 1**A and **1**B). The lower IgG level in naïve HD patients was the result of a slower kinetics of antibodies induced by the vaccines as compared to naïve HC and previously infected participants (**Fig 1**C). Of note, some previously infected HD patients produced higher amounts of antibodies compared to HC (**Fig 1**A), a finding that is consistent with a more severe COVID-19 course in these patients [36–38]. Plasma samples from the 13 PD patients were collected only after the second vaccine dose and all the participants showed detectable S-specific IgG with average plasma levels that were comparable to those of HC (**Fig 1**A and **1**B). S-specific IgG levels highly correlated with RBD-specific IgG, suggesting that, similarly to natural infection, antibodies to this domain dominated the response induced by vaccination in both patients and controls (**Fig 1**D and **S2 Fig**, panel a). Finally, we observed that two vaccine doses induced, in the majority of the participants, undetectable levels of IgM and detectable levels of IgA specific for SARS-CoV-2 S, which were higher in previously infected HC and HD participants vaccinated with BNT162b2 and in naïve HD patients vaccinated with mRNA-1273 compared to naïve HD vaccinated with BNT162b2, and correlated with the corresponding IgG levels (**Fig 1E–1**G and **S2 Fig**, panels b and c).

### Poor neutralization of Wuhan prototypic SARS-CoV-2 and related risk factors in naïve dialysis patients

**Fig 2:**
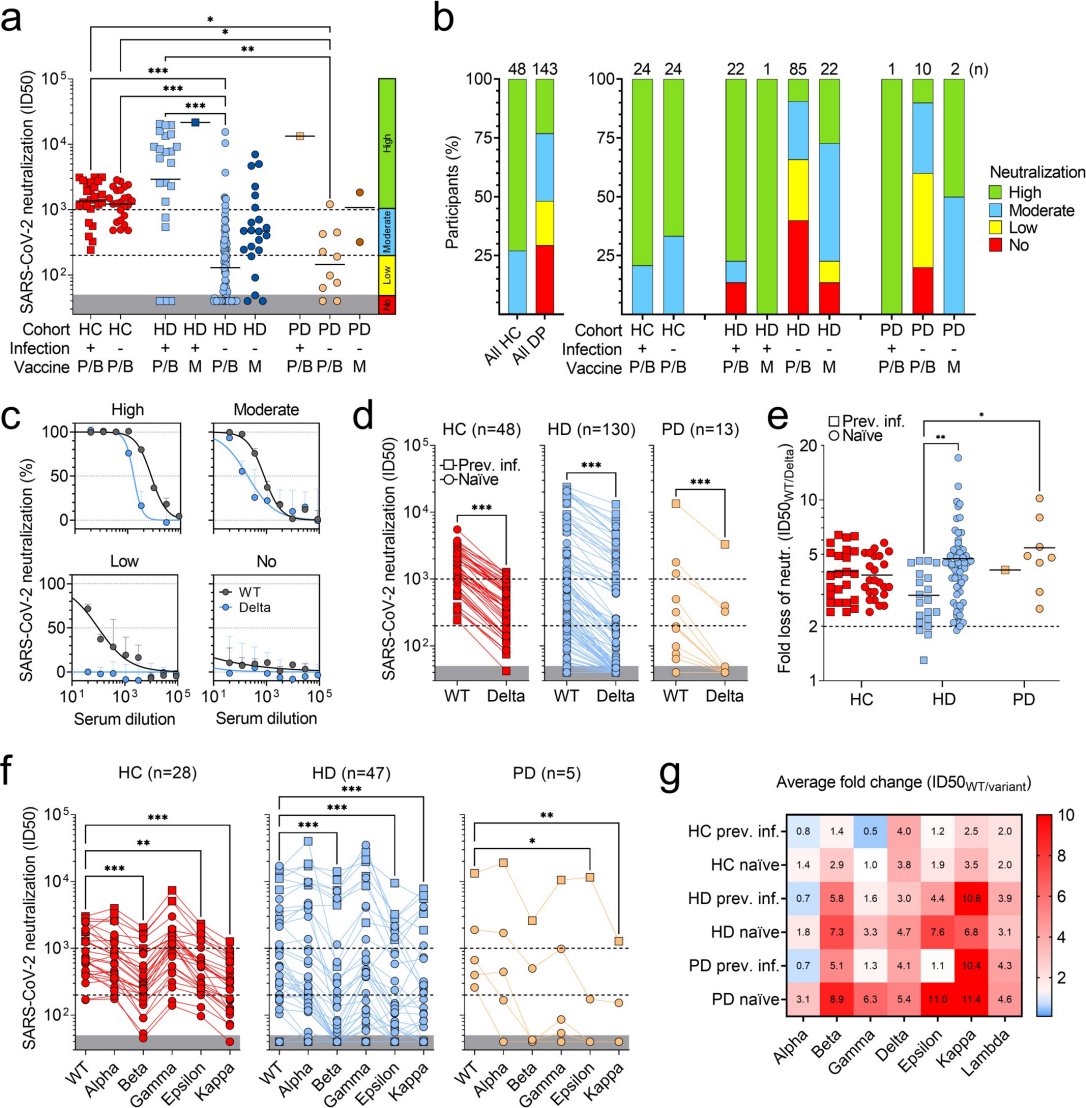
Analysis of neutralizing activity of plasma antibodies against wild-type SARS-CoV-2, Delta and other variants. a) Neutralizing antibody titers (ID50, 50% of inhibitory dilution) against pseudotyped VSV viruses harboring wild-type (D614) SARS-CoV-2 S determined using plasma from previously infected (square) and naïve (circle) healthy controls (HC), hemodialysis (HD) and peritoneal dialysis (PD) patients after two doses of Pfizer/BioNTech (P/B) or Moderna (M) vaccines. Grey areas indicate non-neutralizing titers (<50). A cut-off of 200, determined from the lowest neutralizing titers in HC, and a cut-off of 1’000, determined from the 25% percentile of titers in previously infected HC cohort, were used to distinguish low (50–200) from moderate (200–1’000) or high (>1’000) neutralizing titers. Statistical significance is set as P<0.05 and P-values are indicated with asterisks (* = 0.033; ** = 0.002; ***<0.001). Shown are data from n = 2 independent experiments. b) Percentages of participants with high, moderate, low or no plasma neutralizing antibodies to WT SARS-CoV-2 S after two doses of mRNA vaccine. Total number of participants within each cohort of HC and dialysis patients (DP) and within each subgroup is shown at the top of each bar. c) Neutralization of WT (black) and Delta (B.1.617.2, blue) SARS-CoV-2 pseudotyped VSV by four representative plasma samples showing high, moderate, low or no neutralization to WT SARS-CoV-2. d-e) Side-by-side comparison (d) and fold change analysis (e) of neutralizing titers against WT and Delta SARS-CoV-2 in 48 HC, 130 HD and 13 PD patients. Fold change is calculated as the ratio of ID50 value of WT and ID50 value of Delta variant. Shown are data from n = 2 independent experiments. f) Side-by-side comparison of neutralizing titers against WT and Alpha (B.1.1.7), Beta (B.1.351), Gamma (P.1), Epsilon (B.1.429) and Kappa (B.1.617.1) SARS-CoV-2 variants in 28 HC, 47 HD and 5 PD patients. g) Fold change analysis of neutralizing titers against different variants. Numbers in each cell of panel g indicate the average fold change values of WT ID50 to variant ID50 in all the cohorts analyzed including only participants with ID50 neutralizing titers against WT SARS-CoV-2 greater than 80. Shown are data from n = 2 independent experiments.

To determine the neutralizing activity of vaccine-induced antibodies, we used plasma samples to perform an *in vitro* neutralization assay with pseudotyped vesicular stomatitis virus (VSV) that expresses the Wuhan wild-type (WT, D614) SARS-CoV-2 S glycoprotein (**Fig 2**A and **S3 Fig**, panel a). Compared to HC, 49% of dialysis patients had no or low neutralizing activity against the vaccine-matched SARS-CoV-2 strain (**Fig 2**B). In particular, while most of previously infected HD patients (87%) had moderate to high neutralizing antibody titers, which were similar or higher than those of HC, naïve HD patients had a heterogenous response with 57% of them characterized by a poor neutralizing activity, which was completely absent in the majority (60.7%) of these patients (**Fig 2**A). Of note, the fraction of non-responding patients was higher in naïve HD patients immunized with BNT162b2 (65.9%) than those immunized with mRNA-1273 (22.7%) (**Fig 2**B). Despite higher plasma S-specific IgG titers (**Fig 1**A and **1**B), 60% of naïve PD patients had a similar poor neutralizing response compared to naïve HD patients, with 33.3% of those, who received BNT162b2 vaccine, showing no neutralizing activity (**Fig 2**A and **2**B). The poor neutralizing activity was confirmed also by the low capability of plasma antibodies to inhibit binding of RBD to human ACE2 receptor (**S3 Fig**, panel b). In some previously infected dialysis patients, we observed neutralizing and ACE2-inhibiting antibody titers that were higher than those of HC and correlated with S- and RBD-specific IgG levels (**Fig 2**A and **S3 Fig**, panels c-e).

We next performed a sub-analysis to understand which socio-demographic and clinical data were associated to a significant risk for dialysis patients of being poor responders, here defined as having low or no neutralizing antibody titers after two mRNA-vaccine doses. Among all patients, dialysis mode (HD or PD) was not identified as risk factor, whereas vaccination with BNT162b2 and no previous SARS-CoV-2 infection represented the major risk factors for having a poor neutralizing antibody response (relative risk of 2.75 and 4.50, 95% confidence interval [CI] of 1.37 to 6.30 and 1.78 to 13.1, respectively) (**Table 2**). Within the larger naïve HD group, socio-demographic factors, including gender, age, body mass index, smoke and dialysis features could not be identified as risk factors (**Table 3**). Among comorbidities, we found that patients with heart failure history had an 1.68-increased relative risk of poor response (95% CI, 1.16 to 2.17). As expected, patients in therapy with immunosuppressive drugs had a 1.53 relative risk of poor response to the vaccine (95% CI, 1.05 to 2.02) with, in particular, calcineurin inhibitors accounting for the highest relative risk (1.87; 95% CI, 1.18 to 2.29). Vaccination with BNT162b2 was confirmed as a major factor with a relative risk of poor response of 2.88 (95% CI, 1.47 to 6.55), which increased to 3.60 (95% CI, 1.79 to 8.23) in 22 naïve HD patients who were matched to 22 naïve HD patients immunized with mRNA-1273 by age, gender, dialysis vintage and comorbidities. Among HD patients immunized with BNT162b2, those older than 80 and with heart failure history had an increased risk of poor response of 1.57 (95% CI, 1.18 to 2.08) and 1.55 (95% CI, 1.11 to 1.96), respectively (**Table 3**).

**Table 2:** Analysis of relative risk of poor neutralizing response to COVID-19 vaccination in dialysis patients.

Variable	Analyzed group	Reference group	n_an_	n_ref_	Relative Risk	95% CI	P-value	Significant^§^
**Dialysis mode**	HD/HDF	PD	130	13	1.07	0.661–2.16	>0.9999	ns
**Previous SARS-CoV-2 infection**	No	Yes	119	24	4.50	1.78–13.1	<0.001	***
**mRNA vaccine**	BNT162b2	mRNA-1273	118	25	2.75	1.37–6.30	0.002	**

CI, confidence interval.

^§^ns = non-significant; * = 0.033

** = 0.002

***<0.001.

**Table 3:** Analysis of relative risk of poor neutralizing response to COVID-19 vaccination in naïve HD patients.

	Variable	Analyzed group	Reference group	All naïve HD patients						Naïve HD patients immunized with BNT162b2					
				n_an_	n_ref_	Relative Risk	95% CI	P-value	Significant^5^	n_an_	n_ref_	Relative Risk	95% CI	P-value	Significant^5^
**Socio-demographic data**	**Gender**	Female	Male	36	70	1.07	0.738–1.50	0.8367	ns	30	54	1.11	0.789–1.51	0.6338	ns
	**Age**	>80 years	<80 years	46	60	1.00	0.702–1.39	0.8456	ns	25	59	1.57	1.18–2.08	0.0054	**
	**Body Mass Index (BMI)**	<18.5	18.5–24.9	4	40	1.90	0.934–2.67	0.1217	ns	3	32	1.68	0.715–2.37	0.279	ns
		25–29.9	18.5–24.9	36	40	0.95	0.606–1.48	>0.999	ns	29	32	1.05	0.687–1.58	>0.999	ns
		>30	18.5–24.9	26	40	1.25	0.811–1.87	0.3224	ns	20	32	1.26	0.833–1.87	0.3701	ns
	**Smoker**	Yes	No	15	91	1.36	0.864–1.84	0.2604	ns	14	70	1.25	0.811–1.65	0.361	ns
**Dialysis features**	**Residual diuresis**	Oligoanuria	Non-oligoan.	61	45	1.11	0.791–1.59	0.692	ns	50	34	1.02	0.747–1.44	>0.999	ns
	**Dialysis vintage**	>5 years	<5 years	43	63	1.20	0.852–1.67	0.3231	ns	34	50	1.06	0.755–1.44	0.8171	ns
	**Dialysis mode**	HDF	HD	72	34	1.42	0.969–2.23	0.0942	ns	59	25	1.13	0.815–1.71	0.6165	ns
	**HD duration**	>4 hours	<4 hours	70	35	1.26	0.885–1.93	0.2186	ns	55	28	1.14	0.828–1.68	0.4704	ns
	**Vascular access**	Graft/catheter	Native fistula	104	2	0.56	0.470–1.64	0.504	ns	82	2	0.65	0.138–1.90	0.5425	ns
	**Treatment adequacy (Kt/V)**	<1.2	>1.2	10	82	0.71	0.295–1.29	0.5034	ns	8	66	0.75	0.318–1.24	0.44	ns
	**Anticoagulant therapy**	Yes	No	102	4	1.14	0.636–3.82	>0.999	ns	81	3	0.98	0.651–3.18	>0.999	ns
**Comorbidities**	**Heart failure**	Yes	No	15	91	1.68	1.16–2.17	0.0119	*	14	70	1.55	1.11–1.96	0.0281	*
	**Coronary heart disease**	Yes	No	33	73	1.28	0.899–1.76	0.2053	ns	28	56	1.24	0.886–1.66	0.2302	ns
	**Autoimmune disease**	Yes	No	7	99	1.01	0.436–1.58	>0.999	ns	6	76	1.02	0.453–1.48	>0.999	ns
	**Hypertension**	Yes	No	88	18	0.91	0.643–1.49	0.7962	ns	69	15	0.98	0.707–1.60	>0.999	ns
	**Peripheral arterial disease**	Yes	No	25	81	0.99	0.631–1.40	>0.999	ns	22	63	0.96	0.630–1.33	>0.999	ns
	**Diabetes mellitus**	Yes	No	49	57	1.33	0.952–1.88	0.117	ns	39	45	1.29	0.942–1.79	0.167	ns
	**Pulmonary disease**	Yes	No	35	71	1.09	0.754–1.52	0.6801	ns	28	56	0.97	0.668–1.33	>0.999	ns
	**Cerebrovascular disease**	Yes	No	12	94	1.21	0.689–1.70	0.5464	ns	10	74	0.89	0.302–2.12	>0.999	ns
	**Neurodegenerative disease**	Yes	No	4	102	1.34	0.531–1.88	0.6312	ns	4	84	1.15	0.458–1.60	>0.999	ns
	**Oncologic disease**	Yes	No	11	95	1.33	0.774–1.82	0.3424	ns	8	76	1.16	0.624–1.58	0.7084	ns
	**Psychiatric disorder**	Yes	No	9	97	0.57	0.203–1.14	0.1719	ns	8	76	0.55	0.198–1.05	0.1176	ns
	**Liver disease**	Yes	No	11	95	1.33	0.774–1.82	0.3424	ns	11	73	1.13	0.661–1.54	0.7405	ns
	**Gastrointestinal disease**	Yes	No	22	84	0.96	0.589–1.38	>0.999	ns	18	66	1.02	0.653–1.41	>0.999	ns
	**Kidney transplant**	Yes	No	8	98	1.62	0.956–2.08	0.1339	ns	8	76	1.39	0.821–1.77	0.2526	ns
	**Charlson comorbidity index**	>8	<8	57	49	1.38	0.983–2.01	0.078	ns	44	40	1.36	0.994–1.94	0.0679	ns
**Therapies**	**Oral anticoagulants**	Yes	No	21	85	1.12	0.715–1.57	0.631	ns	16	68	1.06	0.668–1.45	>0.999	ns
	**Antiaggregant drugs**	Yes	No	69	37	1.16	0.819–1.72	0.5378	ns	58	26	1.00	0.734–1.47	>0.999	ns
	**ACE inhibitors/ARBs** ^**1**^	Yes	No	50	56	1.12	0.799–1.57	0.559	ns	14	70	1.09	0.791–1.50	0.6507	ns
	**Calcium antagonists**	Yes	No	42	64	0.82	0.559–1.16	0.3183	ns	31	53	0.77	0.512–1.06	0.1546	ns
	**Beta blockers**	Yes	No	66	40	1.31	0.919–1.96	0.1609	ns	55	29	1.18	0.852–1.75	0.3471	ns
	**Statins**	Yes	No	58	48	1.08	0.775–1.54	0.6964	ns	46	38	0.99	0.724–1.38	>0.999	ns
	**Oral antidiabetic drugs**	Yes	No	17	89	1.18	0.728–1.64	0.5957	ns	14	70	1.25	0.811–1.65	0.361	ns
	**Insulin**	Yes	No	30	76	1.17	0.804–1.62	0.5144	ns	24	60	1.22	0.855–1.63	0.3137	ns
	**Immunosuppressive drugs** ^**2**^	Yes	No	19	87	1.53	1.05–2.02	0.0405	*	18	66	1.25	0.852–1.66	0.2712	ns
	**Oral/intravenous steroids**	Yes	No	12	94	1.38	0.839–1.87	0.2238	ns	13	71	1.21	0.765–1.62	0.5276	ns
	**Calcineurin inhibitors** ^**3**^	Yes	No	7	99	1.87	1.18–2.29	0.018	*	6	78	1.59	0.955–1.93	0.0886	ns
	**Antibiotics**	Yes	No	12	94	0.87	0.434–1.37	0.7595	ns	12	72	0.74	0.367–1.15	0.3252	ns
	**Antiviral drugs**	Yes	No	3	103	0.58	0.107–1.43	0.578	ns	3	81	0.50	0.092–1.23	0.2727	ns
	**Vitamin D derivatives**	Yes	No	83	23	1.39	0.903–2.41	0.163	ns	66	18	1.23	0.848–2.07	0.4033	ns
	**Nonsteroidal anti-inflam. drugs**	Yes	No	4	102	1.34	0.531–1.88	0.6312	ns	2	82	1.55	0.525–1.86	0.5425	ns
**Vaccinations and infections**	**mRNA vaccine**	BNT162b2	mRNA-1273	84	22	2.88	1.47–6.55	0.0005	***	-	-	-	-	-	-
	**mRNA vaccine (matched)** ^**4**^	BNT162b2	mRNA-1273	22	22	3.60	1.79–8.23	0.0002	***	-	-	-	-	-	-
	**Flu vaccination**	No	Yes	19	86	1.04	0.631–1.49	>0.999	ns	17	66	0.99	0.617–1.38	>0.999	ns
	**HBV vaccination**	No	Yes	30	56	1.18	0.798–1.69	0.494	ns	26	43	1.16	0.812–1.62	0.4381	ns
	**HBV vaccine response**	Non-responder	Responder	15	40	1.40	0.812–2.22	0.2393	ns	10	32	1.69	1.04–2.54	0.0608	ns
	**Non-SARS-CoV-2 infections**	Yes	No	42	64	1.17	0.825–1.62	0.4261	ns	35	49	1.01	0.717–1.37	>0.999	ns
	**Non-SARS-CoV-2 resp. infection**	Yes	No	5	101	1.44	0.667–1.94	0.3854	ns	6	78	1.24	0.574–1.65	0.655	ns
	**HBV infection**	Yes	No	16	90	0.99	0.571–1.46	>0.999	ns	15	69	0.90	0.525–1.29	0.7655	ns
	**HCV infection**	Yes	No	5	101	1.44	0.667–1.94	0.3854	ns	4	84	1.57	0.790–1.89	0.2932	ns
	**Bacteriemia/Fungemia**	Yes	No	16	90	1.13	0.676–1.59	0.7854	ns	12	72	1.02	0.586–1.43	>0.999	ns

^1^ACE, angiotensin-converting enzyme. ARB, angiotensin-receptor blocker.

^2^include any of steroids, cyclosporine, tacrolimus, rituximab, mycophenolate mofetil and azathioprine.

^3^include cyclosporine and tacrolimus.

^4^patients were matched by age (±5 years), gender, dialysis vintage (±3 or >10 years), comorbidity index (±3)

^5^ns = non-significant; * = 0.033; ** = 0.002; ***<0.001.

### Loss of neutralization of Delta and other SARS-CoV-2 variants in dialysis patients

We next addressed the question whether vaccine-induced antibodies that neutralize wild-type SARS-CoV-2 could also neutralize current circulating SARS-CoV-2 variants, in particular the Delta variant (B.1.617.2 lineage) (**Fig 2**C). Most dialysis patients (76.9%) had plasma antibodies with low or undetectable neutralizing activity against the Delta variant, with 48.0% and 80.0% of naïve HD and PD patients with detectable levels of neutralizing antibodies against WT SARS-CoV-2 showing complete loss of neutralization against this variant (**Fig 2**D and **S4 Fig**, panel a). Almost all the participants, including HC, had a greater than 2-fold reduction in the neutralizing activity against Delta variant, with naïve HD patients showing up to 17-fold (average 4.7) loss in neutralization, as compared to previously infected patients that showed a maximum 5-fold loss (average 3.0) (**Fig 2**E). Similarly, naïve PD patients showed up to 10-fold (average 5.4) loss of neutralization against Delta variant (**Fig 2**E).

The loss of neutralization against some of the other circulating SARS-CoV-2 variants was higher compared to Delta, in particular for naïve HD patients, who showed an average 7.3-, 7.6- and 6.8-fold reduction in their neutralizing activity against Beta (B.1.351), Epsilon (B.1.429) and Kappa (B.1.617.1) variants (**Fig 2**F and **2**G and **S4 Fig**, panels b-d). Neutralization of Alpha (B.1.1.7), Gamma (P.1) and Lambda (C.37) was also reduced in HD patients, but with minor loss compared to Delta (1.8-, 3.3- and 3.1-fold reduction, respectively) (**Fig 2**F and **2**G and **S4 Fig**, panels e-h). Previously infected HD patients and naïve PD patients also showed a drastic loss of neutralization against different variants, which was comparable to that of naïve HD patients, but higher than that of HC (**Fig 2**F and **2**G and **S4 Fig**). Complete loss of neutralization of Beta, Epsilon and Kappa variants was observed in 37–43.3% of HD and 75% of PD patients with detectable neutralizing antibodies against WT SARS-CoV-2 (**S4 Fig**, panel a).

### The reduced neutralizing activity correlates with a low antibody avidity

**Fig 3:**
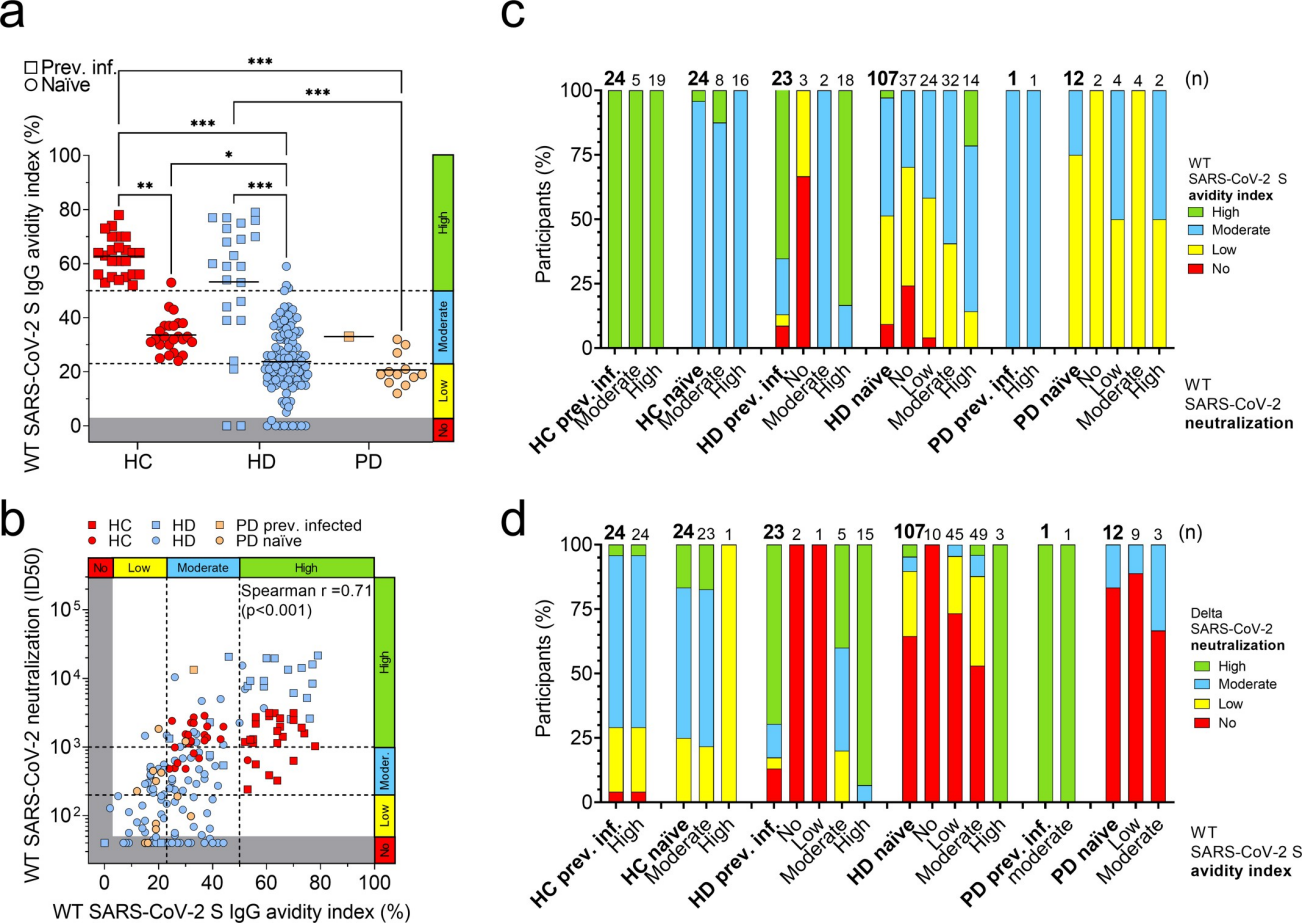
Role of avidity of plasma antibodies in the neutralization of wild-type SARS-CoV-2 and Delta variant. a) Avidity index of plasma antibodies to WT SARS-CoV-2 S in previously infected (square) and naïve (circle) healthy controls (HC), hemodialysis (HD) and peritoneal dialysis (PD) patients after two doses of mRNA vaccine. Grey areas indicate no avidity measured of plasma antibodies with ED50 titers lower than 50. A cut-off of 23%, determined from the lowest avidity index in naïve HC, and a cut-off of 50%, determined from the lowest avidity index in previously infected HC cohort, were used to distinguish low (0–23%) from moderate (23–50%) or high (>50%) avidity. Statistical significance is set as P<0.05 and P-values are indicated with asterisks (* = 0.033; ** = 0.002; ***<0.001). Shown are data from n = 2 independent experiments. b) Correlation analysis between plasma IgG avidity index to WT SARS-CoV-2 S and neutralization of WT SARS-CoV-2 in all the plasma samples collected after the second vaccine dose. c) Percentages of participants having plasma antibodies with high, moderate, low or no avidity to WT SARS-CoV-2 S after two doses of mRNA vaccine. Participants are shown as a total (bold) or divided by level of neutralization of WT SARS-CoV-2 (no, low, moderate, high). Total number of participants within each group is shown at the top of each bar. d) Percentages of participants having plasma antibodies with high, moderate, low or no neutralization of Delta SARS-CoV-2 after two doses of mRNA vaccine. Participants are shown as a total (bold) or divided by level of avidity to WT SARS-CoV-2 S (no, low, moderate, high). Total number of participants within each group is shown at the top of each bar.

To further characterize the poor neutralizing activity of vaccine-induced antibodies, we determined the avidity of plasma antibodies by measuring their binding to SARS-CoV-2 S in presence of sodium thiocyanate, a chaotropic agent that induces dissociation of the antibody from the antigen in case of low affinity. While most of the previously infected HD patients (87%) showed moderate to high-avidity antibodies, a high fraction of naïve HD showed low- (42%) or no (9%) avidity antibodies and most of the naïve PD patients (75%) also showed low-avidity antibodies (**Fig 3**A). As expected, antibodies of all HC that were previously infected with SARS-CoV-2 showed high avidity (index >50%), whereas naïve HC showed moderate avidity (23–50%), consistent with the different duration of affinity maturation ongoing in the two groups (**Fig 3**A). The avidity indexes correlated with SARS-CoV-2 S-specific antibody and neutralizing titers (**Fig 3**B and **S5 Fig**, panel a). In particular, we observed larger fractions of HD patients with moderate to high avidity among patients with higher neutralizing antibody titers (**Fig 3**C). The lower avidity titers were paralleled by higher fractions of dialysis patients showing low- or non-neutralizing antibodies against the Delta and other SARS-CoV-2 variants (**Fig 3**D and **S5 Fig**, panels b-f).

### Rapid decay of neutralizing antibody in 7 months after vaccination

**Fig 4:**
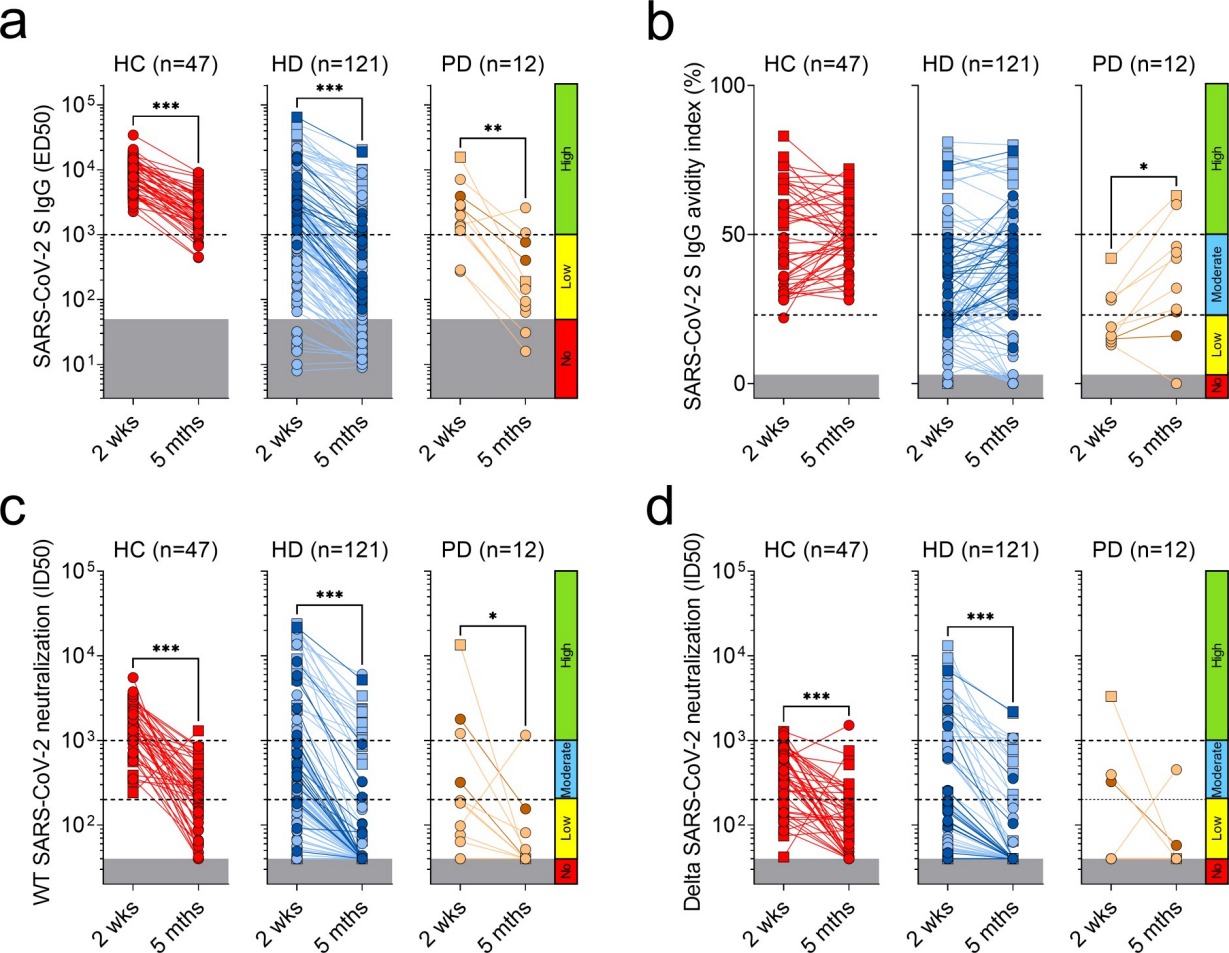
Follow-up analysis of SARS-CoV-2 S IgG, neutralizing titers and avidity index. a) Plasma IgG titers (ED50) to SARS-CoV-2 S after two doses of Pfizer/BioNTech (P/B, red, light blue and light orange) or Moderna (M, dark blue and dark orange) vaccines in samples collected from previously infected (square) and naïve (circle) healthy controls (HC, red), hemodialysis (HD, blue) and peritoneal dialysis (PD, orange) patients 2 weeks and up to 7 months (indicated mean 5 months) after vaccination. Cut-offs are shown as explained in Fig 1. Statistical significance is set as P<0.05 and P-values are indicated with asterisks (* = 0.033; ** = 0.002; ***<0.001). b) Avidity index of plasma antibodies to WT SARS-CoV-2 S. Cut-offs are shown as explained in Fig 3C and 3D) Neutralizing antibody titers (ID50) against pseudotyped VSV viruses harboring wild-type (D614) SARS-CoV-2 S (c) or Delta variant (d). Cut-offs are shown as explained in Fig 2.

We followed 47 HC and 133 dialysis patients (121 HD and 12 PD) up to 7 months after vaccination (mean 5 months) and measured plasma titers and avidity of antibodies to SARS-CoV-2 S and their neutralizing activity against the vaccine-matched SARS-CoV-2 and Delta variant (**Table 4**). We found that 12.8% and 77.4% of HC and dialysis patients had low or undetectable S-specific IgG with a 4.2- and 5.2 fold- average reduction of IgG titers compared to the peak of the antibody response after the second vaccine dose (**Fig 4**A and **S6 Fig**, panel a). This titer decay was paralleled by a 12.7- and 21-fold average reduction of neutralizing antibodies to wild-type SARS-CoV-2 in HC and dialysis patients, which resulted in 40% and 84% of non-responders, respectively (**Fig 4**C and **S6 Fig**, panel c). In the case of antibodies neutralizing the Delta variant, we observed a 6.2- and 9.8-average reduction in HC and dialysis patients, which increased the fraction of non-responders to 72% and 90%, respectively (**Fig 4**D and **S6 Fig**, panel d). In line with the analysis at 2 weeks after the second dose, the most affected patients were those in hemodialysis, naïve to SARS-CoV-2 and vaccinated with BNT-162b2, with 97.5% and 99% of non-responders to WT SARS-CoV-2 and Delta variant, respectively (**Table 4**). Interestingly, only 36.2% and 38.8% of HC and dialysis patients showed an increase in the avidity index of SARS-CoV-2 S IgG, which, however, maintained its correlation with the neutralizing activity (**Fig 4**B and **S6 Fig**, panels b and e). Importantly, despite the overall waning of the antibody response during the follow-up, no new cases of SARS-CoV-2 infection were diagnosed in both HC and dialysis patients after vaccination.

**Table 4:** Follow-up analysis of SARS-CoV-2 S IgG titer, wild-type SARS-CoV-2 and Delta neutralization, and avidity index.

	Nr.	Months after 2^nd^ dose	S IgG titer	Fold reduction		WT neutralization	Fold reduction		Delta neutralization	Fold reduction		Avidity index	Variation (%)	
		Mean (min-max)	Responders (%)^a^	Nr.^b^	Mean (min-max)	Responders (%)^c^	Nr.^d^	Mean (min-max)	Responders (%)^c^	Nr.^d^	Mean (min-max)	Responders (%)^e^	Nr.^f^	Mean (min-max)
**Healthy controls**	**47**	**4.6 (4.5–4.9)**	**41 (87.2)**	**47**	**4.2 (1.6–14.9)**	**28 (59.6)**	**47**	**12.7 (0.2–100)**	**13 (27.7)**	**45**	**6.2 (0.4–28.4)**	**17 (36.2)**	**47**	**1.2 (-29.6–36)**
Prev. infected^P/B^	24	4.7 (4.5–4.9)	24 (100)	24	4.2 (1.6–14.9)	22 (91.7)	24	12.7 (0.2–100)	12 (50)	22	6.2 (0.4–28.4)	5 (20.8)	24	1.2 (-29.6–36)
Naïve^P/B^	23	4.6 (4.5–4.9)	17 (73.9)	23	3.3 (1.6–9)	6 (26.1)	23	4.7 (0.2–17.4)	1 (4.3)	23	2.6 (0.6–8.2)	12 (52.2)	23	-4.4 (-29.6–14)
**Dialysis patients**	**133**	**5.3 (3.4–6.8)**	**30 (22.6)**	**126**	**5.2 (1.6–14.9)**	**22 (16.5)**	**87**	**21.1 (3.4–100)**	**14 (10.5)**	**48**	**9.8 (0.4–28.4)**	**51 (38.3)**	**103**	**7.1 (-12.8–36)**
Hemodialysis	121	5.3 (3.4–6.8)	28 (23.1)	114	10.6 (0.5–83.2)	21 (17.4)	79	11.7 (0.1–100)	13 (10.7)	45	13.3 (1.1–100)	43 (35.5)	93	2.9 (-45.6–40.9)
Prev. infected	21	5.1 (4–6.7)	16 (76.2)	20	9.8 (1.1–72.1)	16 (76.2)	18	10.9 (1–100)	11 (52.4)	18	12 (1.1–100)	5 (23.8)	19	1.9 (-45.6–40.9)
Pfizer-BioNTech	20	5.2 (4–6.7)	15 (75)	19	5.1 (1.8–15.2)	15 (75)	17	9 (1.9–45.4)	10 (50)	17	15.7 (1.1–67)	4 (20)	18	-3.2 (-15.7–7)
Moderna	1	4.0 (4.0–4.0)	1 (100)	1	5.2 (1.8–15.2)	1 (100)	1	9.3 (1.9–45.4)	1 (100)	1	16.4 (1.1–67)	1 (100)	1	-3.7 (-15.7–7)
Naïve	100	5.3 (3.4–6.8)	12 (12)	94	3.4 (3.4–3.4)	5 (5)	61	4.2 (4.2–4.2)	2 (2)	27	3.1 (3.1–3.1)	38 (38)	74	5.3 (5.3–5.3)
Pfizer-BioNTech	80	5.3 (3.4–6.8)	8 (10)	74	10.8 (1.1–72.1)	2 (2.5)	42	11.5 (1–100)	1 (1.3)	14	9.5 (2.2–100)	26 (32.5)	54	3.2 (-45.6–40.9)
Moderna	20	5.5 (3.7–6.2)	4 (20)	20	11 (1.1–72.1)	3 (15)	19	10 (1–100)	1 (5)	13	12 (2.2–100)	12 (60)	20	1.1 (-45.6–40.9)
Peritoneal dialysis	12	5.4 (4.3–6.2)	2 (16.7)	12	9.8 (2.2–20.6)	1 (8.3)	8	14.8 (1.1–59.8)	1 (8.3)	3	6.8 (2.4–37)	8 (66.7)	10	8.8 (-12.5–31)
Prev. infected^P/B^	1	5.3 (5.3–5.3)	0 (0)	1	18.8 (0.5–83.2)	0 (0)	1	19.3 (0.1–100)	0 (0)	1	32.8 (5.6–82.8)	1 (100)	1	12.9 (-13.2–31.4)
Naïve	11	5.4 (4.3–6.2)	2 (18.2)	11	83.2 (83.2–83.2)	1 (9.1)	7	100 (100–100)	1 (9.1)	2	82.8 (82.8–82.8)	7 (63.6)	9	21.2 (21.2–21.2)
Pfizer-BioNTech	9	5.5 (4.8–6)	2 (22.2)	9	12.9 (0.5–30.4)	1 (11.1)	5	7.8 (0.1–23.5)	1 (11.1)	1	7.8 (5.6–9.9)	6 (66.7)	7	12 (-13.2–31.4)
Moderna	2	5.2 (4.3–6.2)	0 (0)	2	14.5 (0.5–30.4)	0 (0)	2	7.1 (0.1–23.5)	0 (0)	1	9.9 (9.9–9.9)	1 (50)	2	14 (-13.2–31.4)
**Total**	180	5.1 (3.4–6.8)	71 (39.4)	173	5.8 (5.1–6.5)	50 (27.8)	134	9.7 (8–11.5)	27 (15)	93	5.6 (5.6–5.6)	68 (37.8)	150	4.9 (0.8–9.1)

^P/B^Pfizer-BioNTech.

^a^ED50>1’000.

^b^participants with ED50 titers >50 at 2 weeks after the second dose were counted.

^c^ID50>200.

^d^participants with neutralizing ID50 titers >80 at 2 weeks after the second dose were counted.

^e^avidity index variation >5%.

^f^participants with detectable titers (ED50>50) at 2 weeks and up to 7 months after the second dose were counted.

## Discussion

In this phase of the COVID-19 pandemic where SARS-CoV-2 variants are rapidly spreading worldwide, we urgently need to understand the efficacy of vaccines administered so far in high-risk immunocompromised populations, in particular patients with chronic diseases [39, 40]. In this study, we provide evidence that dialysis patients do not develop sufficient levels of neutralizing antibodies against SARS-CoV-2 variants after two doses of mRNA vaccines.

The analysis of plasma SARS-CoV-2-specific IgG showed an overall response to mRNA vaccine in 94.4% dialysis patients, which is comparable to data shown in other reports (70.5–96%) [19, 22, 41, 42]. Nevertheless, we observed reduced antibody levels after both the first and the second dose in HD patients who were naïve to SARS-CoV-2, a finding consistent with a delayed immune response in this population [19, 43, 44]. These data suggest that measuring serum antibody titers to SARS-CoV-2 in dialysis patients could help clinicians to identify vaccine non-responders and guide clinical decision-making. However, currently available serological tests detect antibodies against the Spike of the Wuhan SARS-CoV-2 and do not define the level of neutralizing activity against the virus, which is considered a more relevant serological correlate of protection [45].

In this study, we found that 49% of dialysis patients had low or undetectable levels of neutralizing antibodies against the vaccine-matched SARS-CoV-2 with a significant reduction in neutralizing titers against different circulating variants in dialysis patients, which is consistent with an immune evasion by the virus [27, 35, 46]. Based on these data, we calculated that the fraction of dialysis patients non-responding to the vaccine further increased to 77% in the case of the Delta variant, suggesting that the great majority of the patients immunized with two doses of an mRNA vaccine may not develop a protective antibody response against SARS-CoV-2 variants. Importantly, follow-up data collected up to 7 months after vaccination showed a rapid decay of neutralizing antibodies to both wild-type SARS-CoV-2 and Delta variant, which increased the fraction of non-responders to 84% and 90%, respectively. These data suggest that dialysis patients have an increased risk of infection with possible severe outcomes over time from vaccination, an association which has been recently described in a US veteran cohort followed up to 6 months after vaccination [47]. However, we did not diagnose new cases of SARS-CoV-2 infection during the follow-up, which might be explained by the lowest incidence observed in Switzerland in the spring-summer season, and by the strict preventive measures adopted by the dialysis patients that might have compensated for the waning of the vaccine-induced antibodies.

Despite the huge discrepancy between the fraction of non-responders based on antibody levels and their neutralizing activity, we observed a high correlation between these titers, which may help to define proper cut-offs in the serological tests used in clinical settings to identify non-responding patients. Furthermore, the correlation observed between avidity and neutralization suggests that vaccine-induced antibodies require high avidity to neutralize SARS-CoV-2. Therefore, the identification of non-responders may be guided by a SARS-CoV-2 avidity test, as successfully implemented for other infectious diseases [48–52].

We identified that having never been exposed to SARS-CoV-2 is the major factor that increases the risk of a poor antibody response after vaccination, especially in HD patients. Indeed, naïve individuals were shown to develop lower titers of neutralizing antibodies to SARS-CoV-2 variants compared to previously infected individuals after two mRNA vaccine doses [35], indicating that vaccination may boost the neutralizing antibody response in dialysis patients after infection [53]. This effect can be explained by the higher antibody avidity that previously infected patients gained over time compared to naïve patients, who instead showed a delayed and low-avidity response. These findings are consistent with a slower affinity maturation of SARS-CoV-2 S-specific B cells, which appears to be a peculiar immunological feature of the dialysis population.

A recent report by Carr et al [54], showed that naïve HD patients vaccinated with BNT162b2 developed higher neutralizing antibodies against SARS-CoV-2 variants compared to Astra Zeneca—Oxford University AZD1222 vaccine, suggesting that mRNA vaccines provide a higher level of protection in dialysis patients compared to adenovirus-based vaccines. However, a difference in efficacy may exist among mRNA vaccines, as we found that being immunized with BNT162b2 instead of mRNA-1273 was the second most relevant risk factor for a poor response in the dialysis population, especially in naïve HD patients. This difference can be explained by the lower mRNA amount provided by one dose of BNT162b2 compared to mRNA-1273 [11, 12]. Indeed, the mRNA-1273 vaccine was found to be more reactogenic [55] and to induce higher antibody titers in healthy individuals as well as in the more vulnerable elderly population [56–59].

Similarly to other studies analyzing the factors that were associated to low antibody levels after COVID-19 vaccination in dialysis patients, we also found a few risk factors of poor neutralizing antibody response to mRNA vaccines, including age, heart failure and immunosuppression [14, 20, 22, 23, 43, 60–62]. However, other factors, such as gender and dialysis vintage [43], dialysis adequacy measured by Kt/V [20], comorbidities [14] and non-responsiveness to Hepatitis B vaccination [20, 23] were not identified as risk factors in our study.

Our study has some limitations including a missing control group of patients matched by age, gender and comorbidities, without ESKD, while our control group was composed by younger individuals with few or no comorbidities. Another limitation was the low number of PD patients that did not allow to make subgroup analyses of risk factors of poor response. In addition, our patients were unbalanced in terms of type of mRNA vaccine received (BNT162b2 or mRNA-1273), although we had sufficient statistical power to make a comparison, even in a subgroup of patients matched by age, gender and comorbidity index.

In conclusion, our study demonstrates, at the functional level, that mRNA vaccines induce a poor neutralizing and rapidly decaying antibody response against SARS-CoV-2 variants in dialysis patients, in particular in naïve HD patients immunized with BNT162b2. Our findings support the need of an additional boost, preferentially with a high-dose mRNA vaccine, in this population [62–67], which, however, need to be continuously monitored with proper serological tests that measure not only the serum antibody levels, but also their neutralizing activity, either directly or indirectly through an avidity test. Finally, our data suggest that some patients may not respond efficiently even after an additional boost and, therefore, in case of SARS-CoV-2 infection, they should be considered for other therapeutic strategies, including early immunotherapy with monoclonal antibodies.

## Supporting information

S1 Fig Plasma antibody titers to SARS-CoV-2 after one dose of mRNA vaccine. a) Plasma IgG titers (ED50) to SARS-CoV-2 S after one dose of Pfizer/BioNTech (P/B) or Moderna (M) vaccines in previously infected (square) and naïve (circle) healthy controls (HC, red) and hemodialysis (HD, blue) patients. Grey areas indicate non-specific IgG titers <50, a cut-off that was determined on non-specific binding to uncoated ELISA plates. An additional cut-off of 1’000, determined from the lowest titers in previously infected HC, was used to distinguish low (50–1’000) from high (>1’000) IgG titers. Statistical significance is set as P<0.05 and P-values are indicated with asterisks (* = 0.033; ** = 0.002; ***<0.001). b) Percentages of participants with high, low or no plasma IgG to SARS-CoV-2 S after one dose of mRNA-vaccine. Total number of participants within each cohort of HC and dialysis patients (DP) and within each subgroup is shown at the top of each bar.
(TIF)

S2 Fig Kinetics of plasma antibody titers to SARS-CoV-2 in healthy controls and dialysis patients. a) Plasma IgG, IgM and IgA titers to SARS-CoV-2 RBD. b) Plasma IgM and IgA titers (ED50) to SARS-CoV-2 S measured before vaccination (T0), after one (T1) or two (T2) vaccine doses. c) Plasma IgG, IgM and IgA titers to SARS-CoV-2 Nucleoprotein (N).
(TIF)

S3 Fig Correlation of plasma IgG titers, ACE2 inhibition and neutralization against wild-type SARS-CoV-2. a) Neutralization of WT SARS-CoV-2 pseudotyped VSV by four representative plasma samples showing high, moderate, low or no neutralization. b) Inhibition of RBD binding to human ACE2 by plasma antibodies in previously infected (square) and naïve (circle) healthy controls (HC), hemodialysis (HD) and peritoneal dialysis (PD) patients after two doses of mRNA vaccine. Grey areas indicate no ACE2 inhibition by plasma antibodies with BD80 titers lower than 10. A cut-off of 80, determined from the lowest inhibition capacity in HC, was used to distinguish low (10–80) from high (>80) inhibition. Statistical significance is set as P<0.05 and P-values are indicated with asterisks (* = 0.033; ** = 0.002; ***<0.001). Shown are data from n = 2 independent experiments. c) Correlation analysis between plasma IgG titers index and WT SARS-CoV-2 neutralization in all the plasma samples collected after the second vaccine dose. d) Correlation analysis between ACE2 inhibition and plasma RBD IgG titers index in all the plasma samples collected after the second vaccine dose. e) Correlation analysis between ACE2 inhibition and WT SARS-CoV-2 neutralization in all the plasma samples collected after the second vaccine dose.
(TIF)

S4 Fig Comparison of neutralizing antibody titers against wild-type SARS-CoV-2 and other variants. a) Fraction of participants with neutralizing titers against WT SARS-CoV-2 (ID50>40) showing complete loss of neutralization against Alpha (B.1.1.7), Beta (B.1.351), Gamma (P.1), Delta (B.1.617.2), Epsilon (B.1.429), Kappa (B.1.617.1) and Lambda (C.37) variants. b-g) Fold change analysis of neutralizing titers against WT and Beta (b), Epsilon (c), Kappa (d), Alpha (e), Gamma (f) and Lambda (g) SARS-CoV-2 in 28 HC, 36 HD and 5 PD patients with ID50 neutralizing titers against WT SARS-CoV-2 greater than 80. h) Side-by-side comparison of neutralizing titers against WT and Lambda (C.37) SARS-CoV-2 variant in 28 HC, 47 HD and 5 PD patients.
(TIF)

S5 Fig Correlation of avidity and neutralization of plasma antibodies to wild-type SARS-CoV-2 and other variants. a) Correlation analysis between plasma IgG titers index and SARS-CoV-2 S IgG avidity in all the plasma samples collected after the second vaccine dose. b-f) Percentages of participants having plasma antibodies with high, moderate, low or no neutralization of Alpha (b), Beta (c), Gamma (d), Epsilon (e) and Kappa (f) SARS-CoV-2 after two doses of mRNA vaccine. Participants are shown as a total (bold) or divided by level of avidity to WT SARS-CoV-2 S (no, low, moderate, high). Total number of participants within each group is shown at the top of each bar.
(TIF)

S6 Fig Comparison of variation of SARS-CoV-2 S IgG, neutralizing titers and avidity index. a-d) Fold change analysis of SARS-CoV-2 S IgG (a), avidity index (b), neutralizing titers against WT SARS-CoV-2 (c) and Delta variant (d) measured in samples collected at 2 weeks and up to 7 months (average 5 months) after the second vaccine dose. n indicates the number of analyzed participants as explained in Table 4. e) Correlation analysis between plasma IgG avidity index to WT SARS-CoV-2 S and neutralization of WT SARS-CoV-2 in all the 180 plasma samples collected up to 7 months after the second vaccine dose.
(TIF)

S1 File (ZIP)
